# The incidence and prognostic value of HER2 overexpression and cyclin D1 expression in patients with gastric or gastroesophageal junction adenocarcinoma in Israel

**DOI:** 10.3892/ol.2012.1031

**Published:** 2012-11-16

**Authors:** GIL BAR-SELA, DOV HERSHKOVITZ, NISSIM HAIM, ORIT KAIDAR-PERSON, KATERINA SHULMAN, OFER BEN-IZHAK

**Affiliations:** 1 Division of Oncology, Rambam Healthcare Campus, Bat-Galim, Haifa 31096;; 2 Pathology Institute, Technion-Israel Institute of Technology, Technion City, Haifa 32000, Israel

**Keywords:** gastric carcinoma, HER2, cyclin D1, survival, Israel

## Abstract

Human epidermal growth factor 2 (HER2) positivity rates for gastric or gastroesophageal junction (GEJ) adenocarcinoma have been reported at 15–25%. Cyclin D1 (BCL1) is a non-specific proliferative marker. The prognostic significance of HER2 and cyclin D1 is inconclusive, with contradictory data. The aim of this study was to evaluate the incidence of HER2 overexpression in gastric or GEJ patients. The presence of a possible correlation between HER2 status and cyclin D1 staining was assessed; both were evaluated as prognostic markers for survival. The clinical data and histological specimens of 150 consecutive patients diagnosed with gastric or GEJ adenocarcinoma, and treated at our hospital from June 2005 to March 2009, were analyzed. Pathological specimens were immunohistochemically stained for HER2. Immunoreactivity was determined according to the scoring system for gastric carcinoma. Cyclin D1 immunoreactivity was also tested. The results demonstrated that HER2 was positive in 14/150 (9.3%) patients. HER2-positive (HER2^+^) and HER2-negative (HER2^−^) patients did not differ significantly with regard to other clinicopathological parameters. In a multivariate analysis, HER2 positivity was revealed to be a poor prognosis variable (P=0.046; 95% CI, 1.03–3.58). In patients with non-metastatic disease, median survival was 59 months for HER2^−^ and 42 months for HER2+ patients, but this difference was not significant. In patients with metastatic disease, median survival was 9.5 months and 2.5 months for HER2^−^ and HER2+ patients, respectively (P=0.041). Cyclin D1 was not idemonstrated to be a prognostic factor and was not associated with HER2 overexpression. The rate of positive HER2 status in the current group of unselected patients with gastric and GEJ adenocarcinoma was relatively low compared with that observed in the literature. Nevertheless, HER2 positivity was associated with a poor prognosis.

## Introduction

Gastric or gastroesophageal junction (GEJ) adenocarcinoma represents a global health problem. In industrialized countries, the incidence of GEJ and gastric cardial cancers continues to rise, while noncardial gastric cancers have declined over the past few decades. In Israel, the incidence of gastric cancer has remained stable throughout the last decade, annually affecting 650–700 new patients. The incidence trend, described as the age standardized rate (ASR/100,000), is declining in the Jewish population, while rising in the Arab population (1).

The human epidermal growth factor 2 (HER2) gene is a well-described proto-oncogene. High amplification of this gene induces protein overexpression in the cellular membrane, conferring oncogenic properties to a malignant cell (2). Although HER2 gene amplification and protein overexpression have been mostly studied in breast cancer, a wide overexpression variation has been demonstrated in other malignancies (3). HER2 overexpression, measured using immunohistochemistry (IHC), was reported in 4–53% of gastric adenocarcinoma cases, with a median of 18% (4). In a previous study, the incidence of HER2/neu gene amplification was revealed to be higher in GEJ tumors compared with other gastric tumors, and in an intestinal histologic subtype compared with a diffused subtype (5). A recently published systematic review was inconclusive with regard to the prognostic association between HER2 overexpression and poorer survival in patients who underwent curative surgery (4).

The standard of care in advanced gastric carcinoma patients has changed recently, due to a phase III trial adding trastuzumab to chemotherapy in patients with overexpression of HER2 (ToGA trial). The study indicated that this subgroup of patients would benefit from an improvement in overall survival of almost three months (6).

The retinoblastoma (Rb) protein is a tumor suppressor protein that is dysfunctional in many cancers, including gastric adenocarcinoma. One function of phosphorylated Rb (pRb) is to prevent excessive cell growth by inhibiting cell cycle progression in the G1-S checkpoints. Cyclin D1 is a component of this pathway, and it stimulates phosphorylation of the Rb protein by associating with cyclin-dependent kinases (CDKs). Overexpression of cyclin D1 causes pRb pathway dysfunction and stimulation of cell proliferation (7). Upregulation of cyclin D1 has been described in gastric cancer; however, its prognostic value is not fully clear. A possible correlation between HER2 and cyclin D1, both suggested to be poor prognostic tissue markers, had not been examined prior to this study.

The aim of the current study was to evaluate the incidence and the prognostic value of HER2 overexpression among an Israeli population with gastric or GEJ adenocarcinoma. In addition, the correlation between HER2 and cyclin D1 in this patient population was also investigated.

## Materials and methods

### Patients

The records and histological specimens of consecutive gastric or GEJ adenocarcinoma patients who had undergone surgery or biopsies, and were treated in the Division of Oncology at Rambam Healthcare Campus (Haifa, Israel) from June 2005 to March 2009, were evaluated. Clinical and demographical data were retrospectively collected from the computerized hospital medical charts. Data included location of the primary tumor, TNM staging, histological subtype, perineural or vascular invasion, type of surgery, adjuvant chemotherapy and/or radiotherapy, time to recurrence, site of metastases, chemotherapy regimens, response to treatment, time to tumor progression and survival. The study was conducted in accordance with the Helsinki declaration and approved by the Rambam Health Care Campus Ethics Committee, Bat-Galim, Haifa, Israel.

### Immunohistochemical staining

All specimens were fixed in buffered formalin and were immunohistochemically stained for HER2 using the polyclonal antibody anti c-erbB-2 (dilution 1:1400; Dako; Glostrup, Denmark). Immunoreactivity was determined according to the scoring system for gastric carcinoma. For validation, certain cases were evaluated with the Ventana PATHWAY anti-HER-2/neu (4B5) rabbit monoclonal primary antibody (Ventana Medical Systems, Inc., Tucson, AZ, USA). Cases that scored 2+ were further evaluated using the HER2 CISH pharmDx kit (Dako). Additionally, cases were evaluated for cyclin D1 immunoreactivity using the rabbit monoclonal anti-cyclin D1 (SP4) antibody (dilution 1:90; Lab Vision/Neomarkers; Fremont, CA, USA). All immunostaining was performed in the Ventana Benchmark XT immunostainer (Ventana Medical Systems, Inc.). Positive controls, for HER2 (breast cancer) and cyclin D1 (mantel cell lymphoma), and negative controls were run in parallel. A total of 24 biopsy specimens (16%) were compared with surgical specimens.

### Statistical methodology

To identify parameters associated with HER2 overexpression, binary logistic regression was implemented, including calculation of odds ratios (OR) with 95% confidence intervals (CI) and P-values in the bivariate analysis. Survival analysis was performed by the Kaplan-Meier method for overall survival (OS), according to HER2 in locoregional and metastatic diseases. To identify parameters associated with the OS bi-variant, Cox regression was used. P-values, hazard ratios (HR) and 95% CI were presented.

All variables with P<0.2 in bivariate analyses were included in a multivariable explanatory model. A multivariate analysis (Cox regression model) was utilized to determine the independent effects of variables on overall survival. The likelihood ratio was used as a measure of model discrimination.

To analyze the difference between biopsy and surgery histology parameter results, the κ-coefficient was used. A two-tailed P≤0.05 was considered to indicate a statistically significant difference. Statistical analyses were performed with Statistics Products Solutions Services (SPSS) 18.0 software for Windows (SPSS, Inc.; Chicago, IL, USA).

## Results

### General remarks

The key demographic and clinical characteristics of patients are summarized in Table I One hundred and fifty patients, 59% of which were male, were included in the study. The median age at diagnosis was 65 years and 52 (30%) patients were over the age of 70. Arabs constituted 23% of the study population. Poorly differentiated or signet ring cell carcinoma was present in 65% of patients. The disease was metastatic in 68 (45%) patients and signet ring cell carcinoma was the most common histological subtype.

**Figure 1.**
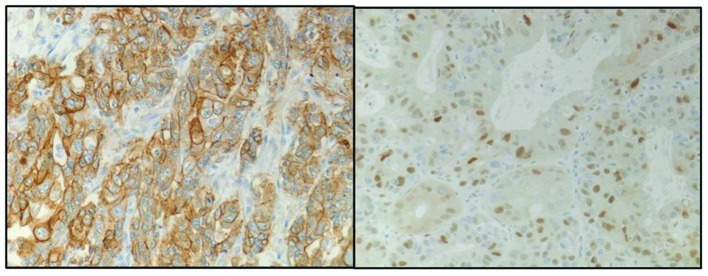
Immunohistochemical analysis. The figure shows two samples, one with positive (+3) membranal staining for human epidermal growth factor 2 (HER2; left panel) and the other with highly positive (70%) staining for cyclin D1 (right panel).

All 150 histological specimens of gastric carcinoma were immunohistochemically stained for HER2 and cyclin D1 immunoreactivity, as described previously (Fig. 1). In 24 cases, the biopsy specimens were compared to the surgical specimens. Only 14 (9.4%) specimens were HER2-positive (HER2^+^). Among the positive specimens, 11 exhibited an IHC HER2 score of +3, while three out of six cases with an IHC score of +2 demonstrated HER2 amplification in the CISH test. All demographic, histopathological and clinical parameters were tested for association with HER2^+^; however, no significant correlation was identified between HER2+ tumors and any of the parameters evaluated (Table I).

### HER2 status in association with stage and survival

HER2^+^ status was identified in 7/82 (8.5%) patients with stage I–III adenocarcinoma who were treated with curative intent, and in 7/68 (10.3%) cases with stage IV adenocarcinoma. In the metastatic group, median survival was 9.5 and 2.5 months for HER2-negative (HER2^−^)and HER2^+^ patients, respectively (P=0.041). First-line therapy consisted of platinum and 5FU/capecitabine, with or without taxanes. No HER2^+^ patients were treated with trastuzumab. In the non-metastatic group, median survival time was 59 months for HER2^−^ and 42 months for HER2^+^ patients, and this difference was not statistically different. In this group, 63 patients were in stage II–III and 19 in stage I of the disease. Adjuvant chemo-radiotherapy was given to 53 out of 63 patients.

### Prognostic factors for survival

A Cox regression model was utilized to determine the independent effects of variables on overall survival. In a bivariate analysis (Table II), stage level was included in the model with P<0.001 (HR, 4.1). The other variables, with P<0.2 in the bivariate analyses, were HER2^+^ (P=0.10; HR, 1.7) and age over 70 years (P=0.19; HR, 1.45). These parameters were also included in the multivariable explanatory model. For illustration of the model, 1- and 2-year mortality were added to Table II. In the Cox regression multivariant analysis, together with stage (stage II–III: HR, 3.38; 95% CI, 1.2–9.5 and stage IV: HR, 1.73; 95% CI, 4.25–32.50), HER2^+^ was also revealed to be an independent negative prognostic factor for survival (HR, 1.2; 95% CI,1.03–3.58; P=0.046), although the positive HER2 group was a small sample size.

### Cyclin D1 and validation tests

Due to the low incidence of HER2^+^ in the study population when the anti-c-erbB2 oncoprotein antibody was used, a sample of 15 cases with an HER2 score of +1 or +2 was also evaluated with the Ventana PATHWAY anti-HER-2/neu (4B5) rabbit monoclonal primary antibody, for validation. Although the staining with the Ventana PATHWAY anti-HER2/neu (4B5) antibody was clearer for evaluation, no difference in the scoring level was observed between the two staining antibodies.

The majority of cases (89.3%) were cyclin D1-positive (>5% staining of tumor cells). Only 16 cases had ≤5% cell staining; all of which were HER2−, and only 2 of which had a weak HER2 staining score of +1. No association was identified between HER2 status and cyclin D1. A threshold of 50% cell staining was used to differentiate between positive and highly positive cyclin D1 staining. No correlation was observed between the level of cyclin D1 positive staining and any of the clinicopathological parameters or survival. Among the 134 positive cases, 71 (47.3%) cases exhibited positive staining (5%<cyclin D1<50%) and 63 (42%) were highly positive (>50% of the tumor cells).

The possibility of a difference in HER2 score or cyclin D1 staining between primary biopsies and surgical specimens was evaluated in 24 cases. No significant differences were observed in HER2 status or score between specimens. Heterogeneity was observed in the cyclin D1 level, while 52% of duplicated cases were not in the same cyclin D1 level group, with a κ value of 0.14 that indicated a slight degree of association.

## Discussion

The prognostic value of HER2 gene amplification and protein overexpression in gastric adenocarcinoma is not as established as it is in breast cancer. In a retrospective study in Singapore, the rate of HER2 overexpression in patients who underwent curative surgery was 9.4% and was inversely correlated with survival in intestinal-type gastric cancer (8). However, a large retrospective study of over 900 cases identified similar rates of positive HER2 cases (<10%), but no correlation with survival was observed (9). A review of 35 published studies, which evaluated the prognostic value of HER2, indicated no differences in the majority of studies, with regard to overall survival (OS). Two studies identified a longer OS, while 13 (37%) observed a significantly poorer OS (4). The current study supports the data indicating that HER2 overexpression in gastric cancer is a poor prognostic sign.

A retrospective study involving 461 consecutive patients with gastric cancer evaluated the clinical and pathological characteristics of gastric cancer in Israel, in comparison to with a Western population. The authors indicated that results unique to the Israeli population were increased incidence of gastric cancer in Ashkenazi Jews and a high incidence of second primary malignancy and family history of cancer. In both study populations, there was no predominance of proximal gastric cancer (10). In the current study, no significant differences were identified between Jewish and Arab populations, or between gastric and GEJ adenocarcinoma patients, with regard to HER2 expression. The median age at diagnosis was similar to that observed in other studies (10,11). However, HER2 positivity in Ashkenazi Jews was not evaluated due to the small number of patients. The rate of HER2 positivity identified in the current study is within the lower range published in the literature (11–13). Tumor heterogeneity is more common in gastric cancer patients than in breast cancer patients, with regard to HER2 scoring. Therefore, a significant number of patients may be missed, as was demonstrated in a previous validation study (12).

Further examination of uncertain cases in relation to tumor heterogeneity, type, grade, location and distribution of polysomy did not provide valuable information for the validation of HER2 status (12). In the current study, all histological specimens were immunohistochemically stained for HER2 using anti c-erB2 oncoprotein antibody (Dako). Several cases were further evaluated by the Ventana PATHWAY anti-HER-2/neu (4B5) antibody. Cases that demonstrated an IHC score of +2 were further evaluated using the HER2CISH pharmDx kit (Dako). This was conducted similarly to the recommendations by Rüschoff *et al*, which suggest that IHC should be the initial testing methodology and FISH/silver *in situ* hybridization should be used to re-evaluate samples with an IHC score of +2 (14).

The clinical significance of cyclin D1 overexpression in gastric cancer is inconclusive (7); it has been demonstrated to be associated with poor differentiation, diffuse type lesions, signet ring cell carcinoma or lymph node involvement, and depth of invasion (15,16). In the present study, the majority of samples were cyclin D1-positive. Even when a threshold of 50% cell staining was applied to differentiate between positive and highly positive cyclin D1 staining, no correlation was identified between cyclin D1-positive staining and survival. Also, no correlation was demonstrated between HER2 status and cyclin D1. There were certain differences in the extent of cyclin D1 staining between the primary biopsy and the full surgical specimen; we propose this was a consequence of heterogeneity for cyclin D1 staining in the full surgical specimen. Therefore, in this case, a primary biopsy specimen taken from a limited location within the tumor may not accurately represent the extent of cyclin D1 staining. Differences in immunostaining between the primary biopsy and the full surgical specimen may also be attributed to differences in fixation between smaller and larger sized tissue samples (17). However, this is less likely in our case, since no differences in the interpretation of HER2 immunostaining were noted.

According to the ToGA trial (a large, multicenter, phase III, randomized controlled trial), trastuzumab in combination with chemotherapy is the new standard option for patients with HER2^+^ advanced gastric and GEJ adenocarcinoma, due to its survival benefit (6). The independent prognostic value of HER2 overexpression, as identified in the current study as well as in others (4), raises the need for clinical evaluation of anti-HER2 monoclonal antibodies as a part of the adjuvant treatment protocol for gastric adenocarcinoma.

In conclusion, the rate of positive HER2 status in the current group of unselected Israeli patients with gastric and GEJ adenocarcinoma was relatively low, compared with that noted in the literature. Nevertheless, positive HER2 was associated with a poor prognosis in Cox regression multivariate analysis.

## Figures and Tables

**Table I.** Associations between patient demographics, clinicopathological parameters and HER2 status in 150 gastric adenocarcinoma cases.

Variable	No. of patients	HER2	Odds ratio	95% confidence interval	P-value
Gender					
Male	89 (59%)	10 (11%)	1.00	Reference	0.34
Female	61 (41%)	4 (6.6%)	0.55	0.16–1.85	
Age (years)					
≤50	27 (18%)	3 (11%)	1.00	Reference	0.13
51–70	71 (47%)	3 (4.2%)	0.35	0.67–1.87	0.22
≥71	52 (35%)	8 (15.4%)	1.45	0.30–6.00	0.60
Ethnicity					
Jewish	116 (77%)	12 (10.4%)	1.00	Reference	0.48
Arab	34 (23%)	2 (6.3%)	0.57	0.12–2.67	
Histology					
Well diff.	10 (7%)	0		0.16	
Moderate diff.	31 (21%)	7 (22.6%)			
Signet ring and poor diff.	96 (65%)	7 (7.2%)			
Mucinous	13 (8%)	0			
Stage					
I	19 (13%)	1 (5.3%)	1.00	Reference	0.80
II and III	63 (42%)	6 (9.5%)	1.90	0.21–16.8	0.56
IV	68 (45%)	7 (10.3%)	2.06	0.24–17.91	0.51
Location					
GEJ and cardia	47 (31%)	5 (10.4%)	1.20	0.38–3.80	0.75
Body	52 (35%)	4 (7.3%)	0.66	0.20–2.25	0.51
Antrum	46 (31%)	5 (10.6%)	1.24	0.39–3.93	0.71
Entire stomach	5 (3%)	0	0.999		
Cyclin D1					
≤5%	15 (10%)	0		0.69	
6–49%	72 (48%)	9 (12.5%)			
≥50%	63 (42%)	5 (8%)			

HER2, human epidermal growth factor 2; diff., differentiated; GEJ, gastroesophageal junction.

**Table II.** Associations between patient demographics, histology parameters and mortality.

Variable	No. of patients	1-year mortality (%)	2-year mortality (%)	Hazard ratio	95% confidence interval	P-value
Gender						
Male	89	39 (43.8%)	51 (57.3%)	1	Ref.	
Female	61	23 (37.7%)	39 (54.1%)	0.75	0.50–1.13	
Age (years)						
≤50	27	7 (26%)	15 (55.6%)	1	Ref.	0.17
51–70	71	25 (35.2%)	36 (50.7%)	0.99	0.57–1.71	0.98
≥71	52	30 (57.7%)	33 (63.5%)	1.45	0.83–2.54	0.19
Stage						
I–III	82	16 (19.5%)	26 (31.7%)	1	Ref.	**>0.0001**
IV	68	46 (67.6%)	58 (85.5%)	4.14	2.76–6.21	
Histology						
Well diff.	10	3 (30%)	3 (30%)	1	Ref.	0.43
Moderate diff.	31	10 (32.3%)	15 (48.4%)	1.14	0.46–2.84	0.77
Signet ring and poorly diff.	97	46 (47.4%)	61 (63%)	1.47	0.64–3.40	0.36
Mucinous	12	3 (25%)	5 (41.7%	0.90	0.30–2.68	0.85
HER2 status						
Negative	136	53 (39%)	74 (54.4%)	1	Ref.	**0.10**
Positive	14	9 (64%)	10 (71.4%)	1.68	0.90–3.15	
Cyclin D1						
≤5%	15	7 (46.7%)	10 (66.7%)	1	Ref.	0.88
6–49%	72	28 (39%)	38 (52.8%)	0.89	0.45–1.76	0.73
≥50%	63	27 (43%)	36 (57%)	0.97	0.49–1.93	0.94

Diff., differentiated.
